# Feasibility, User Acceptance, and Outcomes of Using a Cancer Prehabilitation App for Exercise: Pilot Cohort Study

**DOI:** 10.2196/64427

**Published:** 2025-01-20

**Authors:** Fuquan Zhang, Deepali Bang, Christine Alejandro Visperas, Mon Hnin Tun, San San Tay

**Affiliations:** 1 Department of Rehabilitation Medicine, Changi General Hospital, 2 Simei St 3Singapore, 529889, Singapore, 65 6788 8833; 2 Department of Health Services Research, Changi General Hospital, Singapore

**Keywords:** cancer prehabilitation, mobile app, technology, feasibility, acceptance, cancer, prehabilitation, mHealth, exercise, application, app, mobile application, reliability, smartphone app, sustainability, effectiveness, older patients, older adults, elderly

## Abstract

**Background:**

The efficacy of cancer prehabilitation programs is supported by international reviews and meta-analyses. Technology has been deployed in cancer prehabilitation to address challenges such as access or limited resources. This study evaluated the feasibility, user acceptance, safety, and program outcomes of a newly developed mobile app for cancer prehabilitation. The app integrates with Singapore’s existing health care mobile app, Health Buddy, and provides instructional videos for prescribed exercises.

**Objective:**

The objectives of this study were to investigate the feasibility, user experience, safety, and outcomes of a mobile app for cancer prehabilitation within a hospital-associated, home-based, multimodal cancer prehabilitation program.

**Methods:**

This retrospective study analyzed the records of patients enrolled in the cancer prehabilitation program from September 1, 2022, to March 30, 2023. Patients who participated in the prehabilitation program (n=63) were categorized into 2 groups: those prescribed the app (n=41) and those who were not (n=22). There was further subgroup analysis of those who were prescribed: app users (n=25) versus those who were non-app users (n=16). Demographics, Fried Frailty Phenotype, prehabilitation duration, app use, and functional outcome measures (6-minute walk test [6MWT], 30-second sit-to-stand test [STS], timed up and go test [TUG], and Hospital Anxiety and Depression Scale [HADS]) were collected. Compliance was determined by the completion of prescribed exercises and the accuracy of executing these exercises, with a high compliance rate considered to be at 80% or more. Baseline characteristics and preoperative outcomes were compared between the groups. User satisfaction was assessed through surveys among app users (n=25).

**Results:**

Among 63 patients, 41 (65.1%) patients were prescribed the app, of which 22 (34.9%) patients were users. No significant differences in preoperative functional improvements were observed between app users and nonusers (6MWT: *P*=.60; STS: *P*=.81; TUG: *P*=.53; HADS: *P*=.36), or between those prescribed and not prescribed the app (6MWT: *P*=.94; STS: *P*=.26; TUG: *P*=.39; HADS: *P*=.62). However, high compliance rates (80%) were observed among app users. Patient satisfaction with the app was high (>90%), with positive feedback on ease of use and technical reliability. Baseline measures revealed significantly lower functional scores and higher mean frailty scores in the nonprescribed group.

**Conclusions:**

This preliminary study demonstrates the acceptability, feasibility, and safety of Singapore’s first smartphone app for exercise prescription in cancer prehabilitation. Lower baseline functional outcome measures and a higher mean frailty score in the unprescribed group have implications for the selection process and patient participation. Further studies should include strategies to enhance patients’ readiness for technology, sustainability, and effectiveness in older patients.

## Introduction

Cancer prehabilitation is defined as a set of interventions delivered within the cancer care continuum, occurring during the period between diagnosis and the initiation of definitive treatment [1]. It encompasses baseline assessments of physical function, nutritional status, and psychological well-being. Prehabilitation also involves the identification of potential impairments and the implementation of targeted interventions aimed at optimizing health, thereby reducing the risk and severity of future treatment-related complications. The efficacy of cancer prehabilitation programs is supported by findings from several international reviews and meta-analyses [2-4]. However, ensuring equitable access to prehabilitation services remains a challenge, particularly in settings with limited health care resources or geographical barriers to care [5]. In another study, the preferred method for exercise program delivery was home-based, with 1 supervised session per week [6]. Technology-enabled prehabilitation programs may be able to support home-based programs, which is important to improve adherence and maximize program effectiveness within a short prehabilitation window [5].

A systematic review of cancer prehabilitation programs delivered through technological enablers found the studies to be feasible. While the effectiveness of these programs varied based on the intervention design and the specific technologies used, many studies reported improvements in physical function, activity levels, and overall patient satisfaction. It was recommended that future efforts focus on implementing multimodal programs and using intervention-specific outcome measures to enhance the overall effectiveness of cancer prehabilitation [7].

Our institution has established a hospital-associated, home-based prehabilitation program targeting patients with newly diagnosed gastrointestinal and urological cancers awaiting surgery [8,9]. This multimodal program incorporates medical optimization strategies, individualized exercise prescriptions, and nutritional and mental health interventions. This program was facilitated by a small core team consisting of a coordinator who collects the outcome measures and a physiatrist who sees the patients on the same day as the surgeon visits, providing a 1-stop service. The compliance of the patients to the home exercise program was tracked by phone calls made by the coordinator on a weekly to fortnightly basis. The program has demonstrated significant improvements in functional capacity, as evidenced by metrics such as the 6-minute walk test (6MWT), 30-second sit-to-stand test (STS), and timed up and go test (TUG). There were significant improvements in the Hospital Anxiety and Depression Scale (HADS) as well.

In November 2021, the program integrated a cancer prehabilitation exercise diary into our existing network’s widely used regional health care mobile app, Health Buddy (Singapore Health Services [Singhealth]) [10]. The Health Buddy app allows the user to manage appointments, make payments, and have access to health tools and education.

Despite the wide range of digital health apps [11], no cancer prehabilitation apps are currently available in Singapore. This study aims to evaluate the feasibility, user acceptance, safety, and program outcomes associated with using this mobile app within our multimodal cancer prehabilitation program.

## Methods

### The Cancer Prehabilitation Exercise Diary

**Figure 1.**
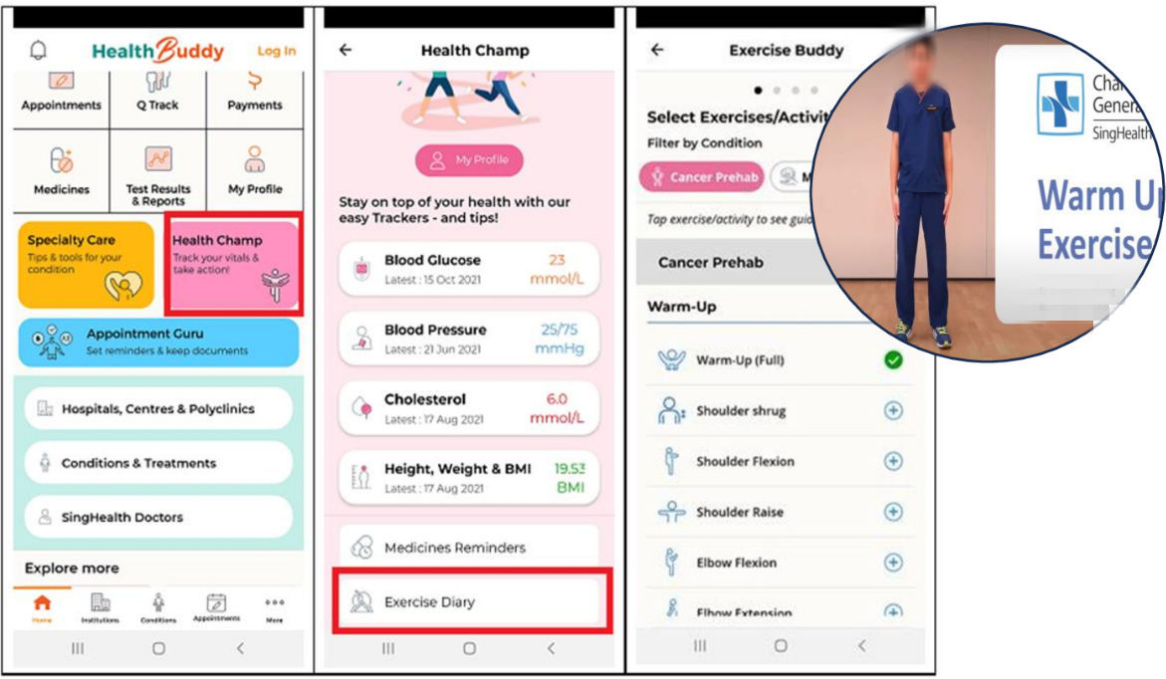
Health Buddy app exercise diary.

The cancer prehabilitation exercise diary incorporates a library of instructional videos demonstrating commonly prescribed exercises. These exercises are selected by a physiatrist and are customized for individual patients to ensure patient safety and efficacy. Patients can use the videos for guidance during their daily exercise routines. Additionally, the diary features functionalities such as customized exercise reminders, exercise logs for tracking progress, and an achievement summary to motivate program adherence (Figure 1). Some patients are sedentary and older patients may not be exercising regularly or making the right movements. The intention of having this exercise diary was to facilitate patients in their exercises as they would be able to view the exercises and follow along. The intention was that it would be easier to follow than figures on paper and it may encourage increased participation. Patients who decline the app will be provided with paper exercise prescriptions whereas those who accepted the exercise prescription on the exercise diary will follow the videos for strengthening exercises in addition to aerobic exercise prescriptions. Both groups of patients were prescribed both aerobic and strengthening exercises.

### Aims and Hypothesis

This study aimed to evaluate the feasibility, user acceptance, safety, and program outcomes associated with using the cancer prehabilitation exercise diary within our multimodal cancer prehabilitation program. The hypothesis was that it is feasible and safe, and having the app will facilitate greater compliance and accuracy of the exercises. Patient satisfaction with the app would be sought. The physical and mental health improvements should be no worse off than the conventional group.

### Study Design

We examined the records of patients who participated in the cancer prehabilitation program from September 1, 2022, to March 30, 2023. The cancer diagnostic groups included colorectal, hepatobiliary, upper gastrointestinal, and urological cancers planned for surgery at our hospital. They were referred by the surgeons at the point of diagnosis to the prehabilitation coordinator.

The inclusion criteria were cancer patients older than 18 years who attended the surgical clinics and were listed for surgery and who were enrolled in the cancer prehabilitation program. Consecutive patients who were enrolled were studied. Patients who declined surgical intervention were excluded from the study. Outcome measures at baseline and the preoperative period were routinely collected, as well as the patient satisfaction surveys for audit purposes.

Participants who received exercise interventions through the cancer prehabilitation exercise diary were categorized into 2 primary groups: those who were prescribed the app and those who were not. Among those prescribed the app, a further subdivision was made into app users and nonusers. The categorization into app prescribed or not prescribed was not decided a priori but based on the physician’s behavior. The categorization of the app used or nonused was also a result of the patient’s autonomous decision. As this is a retrospective study, the participants were nonrandomized. Patients prescribed exercises via the app were invited to complete a patient satisfaction survey upon program completion.

### Demographics

Demographic data, including age, gender, and disease diagnosis, were obtained from patient medical records. Additionally, medical charts were reviewed for data pertaining to the Fried Frailty Phenotype [12] as well as the duration of the prehabilitation program. Information regarding if the patients were prescribed the app, and the reason if not, as well as the actual use of the app were collected.

The Fried Frailty Phenotype [13] assesses physical frailty through 5 criteria: unintentional weight loss; weakness or poor handgrip strength; self-reported exhaustion; slow walking speed; and low physical activity. Patients would be considered frail if they met the score criteria of at least 2 items, prefrail if they met 1 criteria, and nonfrail if they met none. The duration of prehabilitation is the number of days from enrollment to the preoperative visit (typically held on the day before the surgery).

### Baseline and Preoperative Outcome Measures, Compliance, and Adverse Events

Baseline and preoperative outcome measures were collected at the program’s initiation and completion (preoperative visit), respectively. Physical function was assessed using the 6MWT, STS, and TUG. Mental health outcomes were evaluated using the HADS.

Compliance data were also captured, with full compliance defined as completing all prescribed exercises and demonstrating them correctly at the preoperative visit. Partial compliance was defined as having some inaccuracy in exercise performance or not completing the entire set. Although there is no universal agreed-upon threshold, we considered a compliance rate of 80% to be high. Reasons for not prescribing the app or for nonuse of the app were extracted from electronic medical records.

For patients who used the app, a patient satisfaction survey was administered upon program completion. Questions pertaining to the awareness of the Health Buddy app and the use of the smartphone for features within Health Buddy were asked.

### Statistical Analysis

Categorical variables were presented as proportions or percentages, while continuous variables were described using means and SDs or medians and IQRs. A linear mixed-effects model for repeated measures was used to assess differences in outcome measures between groups (prescribed app vs nonprescribed app and app users vs nonusers). Two-tailed statistical tests with a significance level (α) of 0.05 were used. All statistical analyses were performed using Stata 16 (StataCorp LP) software.

### Ethical Considerations

This study was reviewed and approved by our institutional review board (2021/2751). As all patient information in the dataset was obtained from retrospective records and deidentified, informed consent was waived and allowed for secondary analysis without additional consent. No financial compensation was offered to participants of this study.

## Results

### Demographics and Baseline Outcome Measures

Between September 1, 2022, and March 30, 2023, a total of 66 patients were enrolled in the cancer prehabilitation program. A total of 3 patients dropped out from surgery, leaving 63 patients to be included in the study (Table 1). Of these, 41 (65.1%) patients were prescribed exercise interventions through the cancer prehabilitation app. The remaining 22 patients (34.9%) were not prescribed the app for various reasons, including lack of a smartphone or data plan (n=5), undocumented physician rationale (n=9), patient refusal due to a preference for established methods (n=6), short duration to the operation (n=1), and hearing impairment (n=1). Details regarding the distribution of these reasons are provided in Table S1 in Multimedia Appendix 1.

The majority of participants were of Chinese ethnicity, reflecting the demographics of Singapore. No statistically significant differences were observed in age or gender distribution between the 2 groups (app-prescribed vs nonprescribed). Baseline physical function assessments revealed significantly lower scores in the nonprescribed group on the 6MWT, STS, and TUG (Table 1). The nonprescribed group also demonstrated a higher baseline depression score on the HADS. Patients who were not prescribed the app exhibited significantly weaker hand grip strength, slower gait speed, and a higher mean frailty score.

Among the 41 patients prescribed exercises via the cancer prehabilitation app, 25 (58.1%) were identified as users, while 16 (41.9%) were nonusers. Baseline outcome measures did not reveal significant differences between these groups (Table 2).

Patients were categorized under “undefined” compliance, as there were no data available for collection in view of patients not being contactable/no-show for appointments. Patients who had their operation canceled were not included in the analysis (reasons for cancellation of operation: medically unfit due to new medical condition [n=2], transferred to another hospital for a second opinion [n=1]).

**Table 1.** Baseline characteristics of app-prescribed patients versus nonprescribed patients in outpatient cohort.

	Outpatient (app prescribed)		*P* value
	No (n=22, 34.9%)	Yes (n=41, 65.1%)	
Sex, n (%)			.27
Female	10.0 (45.5)	12.0 (29.3)	
Male	12.0 (54.5)	29.0 (70.7)	
Race, n (%)			.62
Chinese	19.0 (86.4)	35.0 (85.4)	
Malay	2.0 (9.1)	2.0 (4.9)	
Indian	0.0 (0.0)	3.0 (7.3)	
Others	1.0 (4.5)	1.0 (2.4)	
Age (years)			
Mean (SD)	72.1 (8.8)	68.2 (8.9)	.11
Cancer diagnostic groups			.59
Colorectal cancer	7.0 (31.8)	13.0 (31.7)	
Hepatobiliary cancer	9.0 (40.9)	21.0 (51.2)	
Upper gastrointestinal cancer	4.0 (18.2)	3.0 (7.3)	
Urological cancer	2.0 (9.1)	4.0 (9.8)	
Duration of prehabilitation			
Mean (SD)	20.8 (8.9)	20.3 (9.3)	.84
Baseline			
Frailty score			
Mean (SD)	2.3 (1.5)	1.3 (1.2)	0.002^a^
Median (IQR)	2.0 (1.0-3.0)	1.0 (0.0-2.0)	0.003**^a^**
6MWT^b^			
Mean (SD)	267.6 (89.7)	348.0 (88.3)	0.002**^a^**
30-second sit-to-stand test			
Mean (SD)	8.5 (3.7)	10.7 (3.9)	.06
Time up and go			
Mean (SD)	16.3 (10.5)	10.5 (3.9)	0.003**^a^**
Anxiety			
Mean (SD)	2.1 (2.0)	2.3 (2.3)	.71
Depression			
Mean (SD)	2.5 (4.2)	1.2 (1.8)	.08
EQ-5D^c^			
Mean (SD)	68.0 (14.9)	70.2 (20.4)	.66
Anxiety and depression base			
Mean (SD)	4.6 (4.9)	3.5 (3.5)	.31
Maximal hand grip strength (kg)			
Mean (SD)	20.3 (11.8)	27.1 (6.9)	0.001**^a^**
Gait speed less than 0.67 m/s, n (%)			0.002**^a^**
No	13.0 (59.1)	38.0 (92.7)	
Yes	9.0 (40.9)	3.0 (7.3)	
Reduced physical activity, n (%)			>.99
No	15.0 (68.2)	27.0 (65.9)	
Yes	7.0 (31.8)	14.0 (34.2)	
Exhaustion, n (%)			.36
No	15.0 (68.2)	33.0 (80.5)	
Yes	7.0 (31.8)	8.0 (19.5)	
Unintentional weight loss, n (%)			.05
No	11.0 (50.0)	31.0 (75.6)	
Yes	11.0 (50.0)	10.0 (24.4)	
Compliance, n (%)			
Undefined	11.0 (50.0)	8.0 (19.5)	—^d^
Full	8.0 (36.4)	24.0 (58.5)	.04^a^
Partial	3.0 (13.6)	9.0 (21.9)	.14

a Statistically significant values.

b 6MWT: 6-minute walk test.

c EQ-5D: EuroQol group 5-dimensions questionnaire.

d Not applicable.

**Table 2.** Baseline characteristics of users and nonusers of the app in outpatient cohort.

	Outpatient (app used)		*P* value
	No (n=16, 41.9%)	Yes (n=25, 58.1%)	
Sex, n (%)			.08
Female	2.0 (12.5)	10.0 (40.0)	
Male	14.0 (87.5)	15.0 (60.0)	
Race, n (%)			.59
Chinese	14.0 (87.5)	21.0 (84.0)	
Malay	0.0 (0.0)	2.0 (8.0)	
Indian	1.0 (6.3)	2.0 (8.0)	
Others	1.0 (6.3)	0.0 (0.0)	
Age (years)			
Mean (SD)	67.0 (10.5)	69.0 (7.9)	.48
Cancer diagnostic groups			.28
Colorectal cancer	4.0 (25.0)	9.0 (36.0)	
Hepatobiliary cancer	11.0 (68.8)	10.0 (40.0)	
Upper gastrointestinal cancer	0.0 (0.0)	3.0 (12.0)	
Urological cancer	1.0 (6.2)	3.0 (12.0)	
Duration of prehabilitation			.77
Mean (SD)	19.8 (9.2)	20.7 (9.5)	
Baseline			
Frailty score			
Mean (SD)	1.1 (0.9)	1.3 (1.3)	.63
Median (IQR)	1.0 (0.0-2.0)	1.0 (0.0-2.0)	.81
6MWT^a^			
Mean (SD)	362.7 (98.4)	338.6 (81.9)	.40
30-second sit-to-stand test			
Mean (SD)	10.9 (3.3)	10.5 (4.3)	.73
Time up and go			
Mean (SD)	10.6 (5.2)	10.5 (3.1)	.94
Anxiety			
Mean (SD)	1.8 (2.0)	2.6 (2.4)	.26
Depression			
Mean (SD)	0.8 (1.4)	1.4 (2.0)	.37
EQ-5D^b^			
Mean (SD)	72.8 (18.1)	68.6 (22.0)	.53
Anxiety and depression base			
Mean (SD)	2.7 (2.9)	4.0 (3.7)	.23
Maximal hand grip strength (kg)			
Mean (SD)	28.7 (7.8)	26.08 (6.4)	.26
Gait speed less than 0.67 m/s, n (%)			.10
No	15.0 (93.4)	23.0 (92.0)	
Yes	1.0 (6.3)	2.0 (8.0)	
Reduced physical activity, n (%)			.50
No	12.0 (75.0)	15.0 (60.0)	
Yes	4.0 (25.0)	10.0 (40.0)	
Exhaustion, n (%)			>.99
No	13.0 (81.3)	20.0 (80.0)	
Yes	3.0 (18.8)	5.0 (20.0)	
Unintentional weight loss, n (%)			.47
No	11.0 (68.8)	20.0 (80.0)	
Yes	5.0 (31.3)	5.0 (20.0)	
Compliance, n (%)			
Undefined	8.0 (50.0)	0.0 (0.0)	—^c^
Full	4.0 (25.0)	20.0 (80.0)	.001**^d^**
Partial	4.0 (25.0)	5.0 (20.0)	.005**^d^**

a 6MWT: 6-minute walk test.

b EQ-5D: EuroQol group 5-dimensions questionnaire.

c Not applicable.

d Statistically significant values.

### Duration of Prehabilitation

Prehabilitation duration was comparable between the 2 groups (mean of 20.3 d for app-prescribed exercise, mean of 20.8 d for nonprescribed exercise) (Table 1). Among patients prescribed the app, the mean prehabilitation duration was slightly longer for app users (20.7 d) compared with nonusers (19.8 d) (Table 2).

### Preoperative Outcome Measures, Compliance, and Adverse Events

Preoperative outcome measures were compared between patients prescribed the app and those who were not prescribed the app. No statistically significant differences in functional improvements were observed between the groups (Table 3). This finding is consistent with the observation that both groups experienced improvements in functional outcomes and mental health, with no documented adverse events.

**Table 3.** Changes in outcome measures in outpatient cohort between app-prescribed and nonprescribed patients.

Mean changes in outcome	Outpatient (app prescribed)		*P* value
	No (n=22, 34.9%)	Yes (n=41, 65.1%)	
6MWT^a^			
Baseline (mean)	267.6	348.0	—^b^
Pre-op (mean, 95% CI)	24.2 (3.8 to 44.6)^c^	23.3 (9.9 to 36.5)^c^	.94
30-second sit-to-stand test			
Baseline (mean)	8.5	10.7	—
Pre-op (mean, 95% CI)	2.3 (0.6 to 3.9)^c^	1.1 (0.1 to 2.2)^c^	.26
Time up and go			
Baseline (mean)	16.3	10.5	—
Pre-op (mean, 95% CI)	−1.3 (−2.3 to −0.3)^c^	−0.8 (−1.4 to −0.1)^c^	.39
Anxiety			
Baseline (mean)	2.1	2.3	—
Pre-op (mean, 95% CI)	−0.5 (−1.7 to 0.6)	−0.3 (−1.0 to 0.5)	.68
Depression			
Baseline (mean)	2.5	1.2	—
Pre-op (mean, 95% CI)	−0.3 (−1.1 to 0.5)	−0.2 (−0.7 to 0.3)	.84
Anxiety and depression base			
Baseline (mean)	4.6	3.5	—
Pre-op (mean, 95% CI)	−0.9 (−2.6 to 0.6)	−0.5 (−1.6 to 0.5)	.62
EQ-5D^d^			
Baseline (mean)	68.0	70.2	—
Post-op 3 month (mean, 95% CI)	8.8 (−2.3 to 19.8)	−1.6 (−7.9 to 4.7)	.11

a 6MWT: 6-minute walk test.

b Not applicable.

c Statistically significant drop or increase from the baseline.

d EQ-5D: EuroQol group 5-dimensions questionnaire.

Of all patients who were prescribed the app, 24 patients (58.5%) exhibited full compliance, 9 (21.9%) patients exhibited partial compliance, and 8 (19.5%) patients were lost to follow-up. Among those who were not prescribed the app, 8 (36.4%) patients were fully compliant, 3 (13.6%) patients were partially compliant, and 11 patients were lost to follow up (Table 1). The higher incidence of full compliance in the app-prescribed group as compared with the nonprescribed group was statistically significant (*P*=.04).

Among those who were prescribed the app, there was no significant difference in the functional and mental health outcome improvements during the preoperative period between the app users and the nonusers (Table 4).

**Table 4.** Changes in outcome measures between users and nonusers of the app.

Mean changes in outcome	App used		*P* value
	No (n=16, 41.9%)	Yes (n=25, 58.1%)	
6MWT^a^			
Baseline (mean)	362.7	338.6	—^b^
Pre-op (mean, 95% CI)	28.8 (4.8 to 52.8)^c^	21.0 (4.9 to 37.1)^c^	.60
30-second sit-to-stand test			
Baseline (mean)	10.9	10.5	—
Pre-op (mean, 95% CI)	0.9 (−0.9 to 2.9)^c^	1.2 (0.01 to 2.4)	.81
Time up and go			
Baseline (mean)	10.6	10.5	—
Pre-op (mean, 95% CI)	−0.5 (−1.6 to 0.6)^c^	−0.9 (−1.6 to −0.2)^c^	.53
Anxiety			
Baseline (mean)	1.8	2.6	—
Pre-op (mean, 95% CI)	0.4 (−1.0 to 1.8)	−0.6 (−1.6 to 0.4)	.27
Depression			
Baseline (mean)	0.8	1.4	—
Pre-op (mean, 95% CI)	−0.1 (−0.9 to 0.7)	−0.3 (−0.9 to 0.3)	.74
Anxiety and depression base			
Baseline (mean)	2.7	4.0	—
Pre-op (mean, 95% CI)	0.2 (−1.7 to 2.2)	−0.9 (−2.3 to 0.5)	.36
EQ-5D^d^			
Baseline (mean)	72.8	68.6	—
Post-op 3 month (mean, 95% CI)	−5.8 (−17.9 to 6.2)	0.3 (−7.6 to 8.2)	.41

a 6MWT: 6-minute walk test.

b Not applicable.

c Statistically significant drop or increase from the baseline.

d EQ-5D: EuroQol group 5-dimensions questionnaire.

High compliance rates (80%) were observed among app users. Conversely, a high proportion of nonusers (50%) did not return for the preoperative visit or were not contactable. For the remaining nonusers who returned for the pre-op visit and underwent surgery, half (25%) demonstrated full compliance with the exercise program, while the other half (25%) exhibited partial compliance (Table 2). The higher full compliance rate in the app users as compared with the nonusers was statistically significant (*P*=.001).

### User Satisfaction Survey

**Figure 2.**
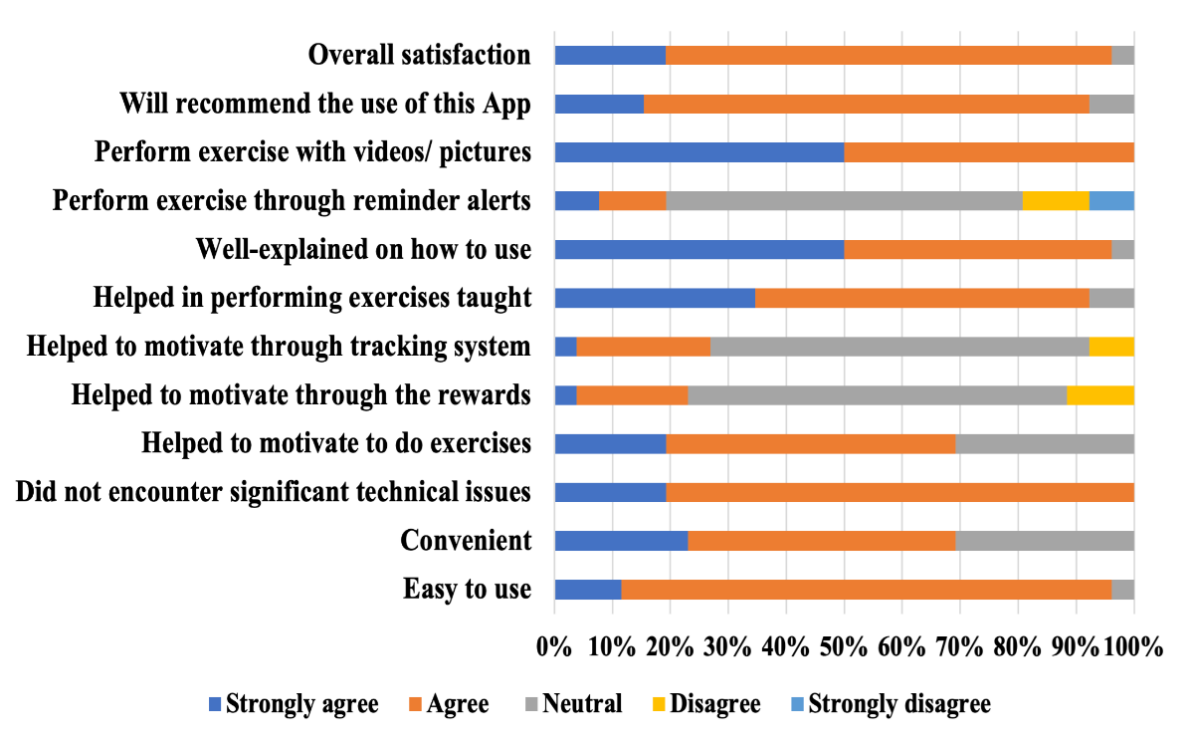
Impression of the prehabilitation smartphone app.

A 76% (19/25) response rate was observed for the patient satisfaction survey among app users. The survey revealed high overall satisfaction with the app, ease of use, and technical reliability, with over 90% (23/25) of respondents reporting these aspects favorably. Furthermore, a high proportion of users indicated a likelihood to recommend the app to others. Patients also reported generally positive experiences with the clarity of exercise instructions and their ability to perform the exercises as instructed (Figure 2).

However, the survey identified the app’s motivational features, particularly reminders and rewards, as an area for improvement. Interestingly, a significant portion of app users reported prior familiarity with the Health Buddy app and regular smartphone use for various activities. A total of 100% (25/25) of responders were aware of the Health Buddy app; 100% (25/25) of responders were aware of the Health Buddy functions; and 80% (20/25) , 64% (16/25), and 60% (15/25) of the responders had used the app for checking or scheduling appointments, receiving health education and making payments respectively. Features such as ordering medication and tracking of clinic queues were less used, at 44% (11/25) and 32% (8/25) respectively.

## Discussion

### Principal Findings

A recent systematic review [13] identified technical barriers, lack of direct supervision, and noncompliance as key challenges in implementing technology-supported cancer prehabilitation programs. Our findings suggest these concerns may not be universally applicable.

Patient satisfaction surveys revealed that app users in our study reported high technical reliability, ease of use, and the ability to follow exercise instructions (Figure 2). This suggests that technical issues were not a significant barrier for our user population. This could likely be attributed to the fact that the exercise diary was incorporated into a well-established health care app in Singapore’s largest health care system [10,11]. As such, familiarity with an existing app might have an important role in facilitating ease of use, making this a more user-friendly experience. Additionally, a curated selection of suitable exercise videos will be made available in a patient’s individualized cancer prehabilitation exercise diary within the Health Buddy app. This user-centric approach streamlines the process, enabling patients to commence their program effortlessly by accessing the designated videos.

Furthermore, a high compliance rate was observed among app users, with 80% achieving full compliance and 20% achieving partial compliance. These findings indicate that a lack of direct supervision did not hinder functional improvement in our program for the population that used the app. Conversely, those who were not prescribed had a higher noncompliance rate and loss of follow-up. That suggests compliance may be an issue with home-based programs itself.

The development of cancer prehabilitation applications is a growing field. For instance, a prototype app designed to support blood flow restriction training and sports nutrition demonstrated high user satisfaction regarding ease of use, utility, and overall function [14]. Our study adds to this emerging body of evidence by investigating the feasibility and user acceptance of a mobile app for cancer prehabilitation program delivery.

### Feasibility and Acceptance of App-Based Prehabilitation

Among the 63 patients in the study, only 41 (65.1%) patients were prescribed exercise interventions through the app. There is borderline acceptance by physicians and patients. Given the full compliance rate of 80% among app users, app-based prehabilitation is feasible.

The main reasons for not prescribing the app included (1) patient limitations: lack of technological knowledge, smartphone/data plan limitations, hearing impairment, or short prehabilitation windows; (2) patient preferences: unwillingness to participate; and (3) undocumented rationale: in 10 cases, the physician’s rationale for not prescribing the app was not documented.

The study highlights the potential impact of socioeconomic factors on technology readiness for cancer prehabilitation. Unlike studies in Western countries with established social acceptance of telehealth [15], a prior local study suggests lower acceptance among diabetic patients [16]. This underscores the need to address potential disparities in access and use. Similar to interventions promoting telehealth for diabetic patients, considerations for app-based prehabilitation might include (1) tailoring support to specific socioeconomic groups less likely to use technology, (2) providing trained staff to address patient concerns and guide app engagement, and (3) exploring the feasibility of loaner devices for the prehabilitation period.

A recent scoping review of reviews on digital health and telehealth in cancer care identified gaps in the literature [12]. These gaps include (1) studies focusing on older adults or those experiencing bereavement, (2) research on the long-term sustainability of telehealth interventions, and (3) comparative studies that directly evaluate telehealth versus in-person interventions.

Similarly, in our patient population, addressing these knowledge gaps is crucial for overcoming challenges associated with nonreadiness and nonacceptance of technology-enabled prehabilitation programs.

### Baseline Differences and Preoperative Outcomes

Patients who were not prescribed the app exhibited significantly lower baseline scores on functional performance measures, and a higher baseline depression score on the HADS. Furthermore, the Fried Frailty Phenotype assessment revealed significantly weaker handgrip strength, slower gait speed, a higher incidence of unintentional weight loss, and a higher mean frailty score in the nonapp group [12].

The Fried Frailty Phenotype focuses on physical frailty [12]. Cognitive frailty, defined by the International Consensus Group organized by the International Academy on Nutrition and Aging and the International Association of Gerontology and Geriatrics, refers to a syndrome in older adults characterized by both physical frailty and cognitive impairment, excluding dementia diagnoses [17]. Our study design did not assess cognitive function, leaving it unclear whether cognitive frailty was present in our cohort and potentially influenced physician or patient selection regarding app prescription.

The significantly higher Fried frailty score in the nonprescribed population suggested that being generally frailer and having lower baseline outcome measures may impair program participation. This is in concurrence with a study demonstrating a negative moderate significant correlation found between frailty and participation [18]. This may be even more so for technology-based programs in the face of a challenging new diagnosis. Participation in this study was a broad concept with several aspects, such as preferences, enjoyment, and satisfaction from activities [19].

Both app users and nonusers who were prescribed the app demonstrated improvements in functional measures (6MWT, STS, and TUG) at the preoperative assessment, with no statistically significant differences observed between these groups. Similarly, no significant differences in preoperative outcomes were found when comparing patients prescribed the app to those who were not. While high compliance was observed among app users who were able to follow the exercise videos, these findings suggest no preoperative outcome advantage associated with app use in this preliminary study. Compliance is defined by the completion of the prescribed set of exercises and the accuracy of the exercises. Using the app may have resulted in greater accuracy of the exercise performed, as was intended.

### Patient Satisfaction and Future Considerations

A high level of user satisfaction was observed, with 90% of postintervention survey respondents reporting satisfaction with the app and a willingness to recommend it to others. This finding suggests promise for the app’s utility among patients receptive to technology-enabled health care interventions.

In a mixed methods systematic review of access, acceptance, and adherence to cancer prehabilitation, there was a perceived value of home-based prehabilitation and the acceptability of tele-prehabilitation [13]. The availability and extent of integrated health care professional supervision and support were perceived to enable intervention access and adherence, especially if this was personalized. Other studies found prehabilitation enabled through technology to be acceptable and perceived to be accessible, especially during the pandemic [6,13,20,21]. Thus, there is value in refining our app-enabled prehabilitation program in the longer term.

Future research efforts should include potential pilot trials among older adults as well as diverse socioeconomic groups, with an aim to adopt strategies tailoring specific support for them while overcoming nonadherence challenges. To promote scalability and integration within our existing health care systems, prioritizing strategies to enhance patient readiness and adoption of technology-based prehabilitation programs should also be considered. Additionally, investigating the long-term sustainability of the intervention within the health care system and staff fidelity to program implementation are crucial areas for further exploration.

### Limitations of Study

One limitation of the study is that we have a substantial number of participants who were eventually uncontactable or lost to follow-up, contributing to the missing data pool. We were unable to determine the reason for this, whether this was due to participant disengagement versus an inability to successfully operate the app. Nonetheless, efforts should be made in future research to implement strategies for real-time monitoring of a patient’s progress and proactive intervention to address potential issues promptly, such as troubleshooting technical problems early or initiating motivational interviewing to address concerns or disinterest early to help maximize retention.

The participating groups were nonrandomized and heterogeneous. As such, selection bias can also be a potential concern in our study.

Finally, given the small sample size, our findings are preliminary and might be limited to acceptability and usability. Further research with a larger sample is needed to confirm and generalize these results.

### Conclusion

In conclusion, this preliminary study demonstrates the acceptability, feasibility, and safety of Singapore’s first smartphone app for exercise prescription in cancer prehabilitation. Future research directions include investigating the program’s effectiveness among older adults, exploring strategies to enhance patient readiness for technology-based interventions, evaluating the program’s long-term sustainability within the health care system, and assessing staff fidelity to program implementation.

## Supplementary material

10.2196/64427 Multimedia Appendix 1 Reasons for not prescribing the app.

## Abbreviations

6MWT: 6-minute walk test
HADS: Hospital Anxiety and Depression Scale
STS: 30-second sit-to-stand test
TUG: timed up and go test
