# Supplementary material for: Feasibility, User Acceptance, and Outcomes of Using a Cancer Prehabilitation App for Exercise: Pilot Cohort Study
Source: JMIR Form Res. 2025 Jan 20;9:e64427. doi: 10.2196/64427 (PMC11769688; doi:10.2196/64427)
Supplement: Multimedia Appendix 1 [file formative-v9-e64427-s001.docx]

**Table S1. Reason for not prescribing the app**

| **Reasons for not prescribing the app** | | **Number of patients** |
| --- | --- | --- |
| 1 | Hearing impaired | 1 |
| 2 | Short duration to op | 1 |
| 3 | Patient not keen | 6 |
| 4 | Not documented | 9 |
| 5 | Not tech-savvy/No data plan/No phone | 5 |
| **Total number of patients not prescribed** | | 22 |
